# Long-term efficacy of ultrasound-guided high-intensity focused ultrasound treatment of breast fibroadenoma

**DOI:** 10.1186/s40349-017-0083-1

**Published:** 2017-03-16

**Authors:** Roussanka Kovatcheva, Katja Zaletel, Jordan Vlahov, Julian Stoinov

**Affiliations:** 1 Department of Thyroid and Metabolic Bone Disorders, University Hospital of Endocrinology, Medical University of Sofia, 2, Zdrave Street, 1431 Sofia, Bulgaria; 2 Department of Nuclear Medicine, University Medical Centre Ljubljana, Ljubljana, Slovenia; 3 Department of Endocrine Surgery, University Hospital of Endocrinology, Medical University of Sofia, Sofia, Bulgaria

**Keywords:** Breast fibroadenoma, High-intensity focused ultrasound (HIFU), Ultrasound (US) guidance, Ablation techniques, Interventional ultrasonography

## Abstract

**Background:**

To assess the long term efficacy and tolerability of one or two ultrasound (US)-guided high-intensity focused ultrasound (HIFU) treatment in patients with breast fibroadenoma (FA).

**Methods:**

Twenty patients with 26 FA were selected for US-guided HIFU. The therapy was performed in one or two sessions. FA volume was assessed before and followed up to 24 months after the last HIFU. After each treatment, adverse events were evaluated.

**Results:**

In 19/26 FA (73.1%) one HIFU was performed (group 1), whereas 7/26 FA (26.9%) received second HIFU (group 2) 6-9 months (median, 7 months) after the first session. In group 1 and 2, FA volume decreased significantly at 1-month (*p* < 0.001) and 3-month follow-up (*p* = 0.005), respectively, and continued to reduce until 24-month follow-up (*p* < 0.001 and *p* = 0.003, respectively). At 24 months, mean volume reduction was 77.32% in group 1 and 90.47% in group 2 (*p* = 0.025). Mild subcutaneous edema was observed in 4 patients and skin erythema in 3 patients.

**Conclusions:**

US-guided HIFU represents a promising non-invasive method with sustainable FA volume reduction and patient’s tolerability. Although one treatment is highly efficient, the volume reduction can be increased with second treatment.

**Trial registration:**

NCT01331954. Registered 07 April 2011.

## Background

Breast fibroadenomas (FA) are the most prevalent benign tumours, accounting for up to 70% of benign breast lesions [1, 2]. Most frequently, they affect females in the reproductive period with two peaks of incidence in the third and in the fifth decade of life. They may also occur after menopause as a result of hormone replacement therapy [3, 4]. Although FA, typically consisting of stromal and epithelial cells, usually occurs unilaterally, multiple lesions in the same breast or bilaterally may arise in 20% of cases [4, 5]. Most patients clinically present with palpable breast lump, detected during medical examination or self-examination, or occasionally with breast pain [2, 6]. Without treatment, a minority of FA decrease in size or disappear, more than half of them remain unchanged, and some of them significantly increase [7]. The long-term risk for breast carcinoma in women with FA has not been established [8, 9] and therefore, a conservative approach to the treatment seems to be safe especially in younger patients [5, 10, 11]. However, some patients prefer elimination due to large size, physical discomfort and anxiety about growth or malignancy. The widely employed surgical excision may result in scar formation, breast volume loss and potential for nipple areolar distortion or displacement [12]. Different non-surgical techniques have been reported. Vacuum-assisted biopsy and cryoablation both demonstrated excellent efficacy, safety and high level of patients’ satisfaction though being minimally invasive [13–15]. The thermoablation procedures such as laser, radiofrequency or microwave ablation have been reported, but they are more frequently applied in breast cancer patients [16–19].

The only non-surgical and non-invasive procedure, employed until today, is high-intensity focused ultrasound (HIFU), where thermal destruction is achieved by precisely delivered energy to a given area in soft tissue without interrupting skin integrity. Although this technique has been applied in the treatment of breast cancer, prostate tumours, uterine fibroids, liver or renal tumours, the experience in breast FA is limited [20]. More than a decade ago, magnetic resonance (MR)-guided HIFU ablation of FA was proposed on the basis of demonstrated feasibility and efficacy in a small group of patients [21]. Recently, Peek et al. showed 43.5% mean FA volume reduction 6 months after circumferential ultrasound (US)-guided HIFU treatment of 19 patients [22]. A multicentre study of Kovatcheva et al. established that US-guided HIFU treatment of 51 FA resulted in 72.5% volume reduction at 1-year follow-up [23]. However, data on prolonged effectiveness and persistence of volume reduction are missing.

In this prospective study, our aim was to assess the long-term treatment efficacy and tolerability of one or two US-guided HIFU sessions in patients with breast FA.

## Methods

### Study design

In our single-centre study, performed between May 2011 and April 2015, from 58 females with clinical suspicion of one or more FA in one or both breasts, 20 symptomatic patients with 26 FA were selected for treatment with US-guided HIFU. The study was approved by the local ethics committee and informed consent was obtained from all individual participants included in the study.

The eligibility criteria included the age of 18 years or more; the clinical diagnosis of breast FA based on palpation, US examination alone for patients < 35 years of age, and mammography in addition for women older than 35 years with Breast Imaging and Reporting Data System (BI-RADS) score ≤2; and a histological confirmation after large-core biopsy using a 16-gauge needle size performed at least two weeks before therapy and verified by two independent readers. The targeted FA size had to be larger than 10 mm, without macrocalcifications inducing a substantial shadowing and situated within the treatable area, which was 5 to 28 mm from the skin surface. The intended size of the ablation unit was 9 mm in length and 1.8–2.5 mm in width and the depth of each ablation unit was adjusted automatically to be centred with the antero-posterior diameter of the target. The rib cage had to be outside the treatment cone or at least 10 mm behind the focal point. Those criteria had to be fulfilled in treatment conditions, once the breast was immobilized and eventually compressed. We excluded women who were pregnant or lactating, those with US suspicions for malignancy, BI-RADS score > 2 or with microcalcifications within the lesion. Also those with history of breast cancer, history of laser or radiation therapy of the targeted breast and those with breast implants were excluded. Thirty-three patients did not meet the inclusion criteria for the following reasons: pregnancy (*n* = 1); unconfirmed diagnosis of FA (*n* = 11); FA inaccessibility (*n* = 21) such as localization partly behind the nipple, close to the rib cage, outside the treatable area, macrocalcifications in the lump; 4 patients refused to participate.

At a selection visit prior to HIFU ablation, body mass index (BMI) was calculated according to the formula: *weight/height* ^*2*^ (kg/m^2^) and US characteristics of FA were evaluated. After the first HIFU, follow-up visits with US assessment of FA were performed at 1, 3 and 6 months. At 6-month follow-up, if FA volume reduced for less than 50% or its absolute volume exceeded 1.5 ml, a second HIFU ablation was performed and all patients were followed-up at 12 and 24 months after the last treatment. Comparison of FA characteristics was conducted on the basis of the number of treatment procedures.

### US evaluation

US evaluation of the targeted FA was performed using an 8-MHz linear probe and a real-time color Doppler US system (Aloka, Prosound Alpha 7, Tokyo, Japan). The first dimension (*d1*) was recorded parallel to the skin in radial position to the nipple. The second dimension (*d2*) was measured orthogonal to *d1*, and the third dimension (*d3*) was measured in anteroposterior direction. The FA volume was calculated by the ellipsoid model (*d1 · d2 · d3 · π/6*). Volume reduction (%) was calculated as: *([basal volume – final volume] · 100)/basal volume*. Before the treatment, the intranodular color flow Doppler (CFD) pattern was evaluated as follows: pattern 0, absence of flow; pattern I, presence of flow with patchy, uneven distribution; pattern II, clearly increased flow with patchy distribution; pattern III, marked increase in blood flow with diffuse homogeneous distribution [24]. US-guided large-core biopsy was carried out in each patient and histological analysis was performed by two independent readers to prove the benign nature of the targeted FA.

### HIFU intervention

HIFU treatment was conducted with a real-time US-guided HIFU system (EchoPulse, Theraclion, Paris, France), described in details previously [23]. The machine consists of a corpus with energy generator, a treatment head and a touch screen interface enabling a procedure planning and monitoring. The treatment head consists of an integrated 7.5 MHz linear array transducer for imaging, a 3 MHz spherical transducer (diameter of 38 or 56 mm and focal region of 0.5x1.0x2.5 mm) to generate HIFU and a cooling system to prevent skin burn.

**Fig. 1:**
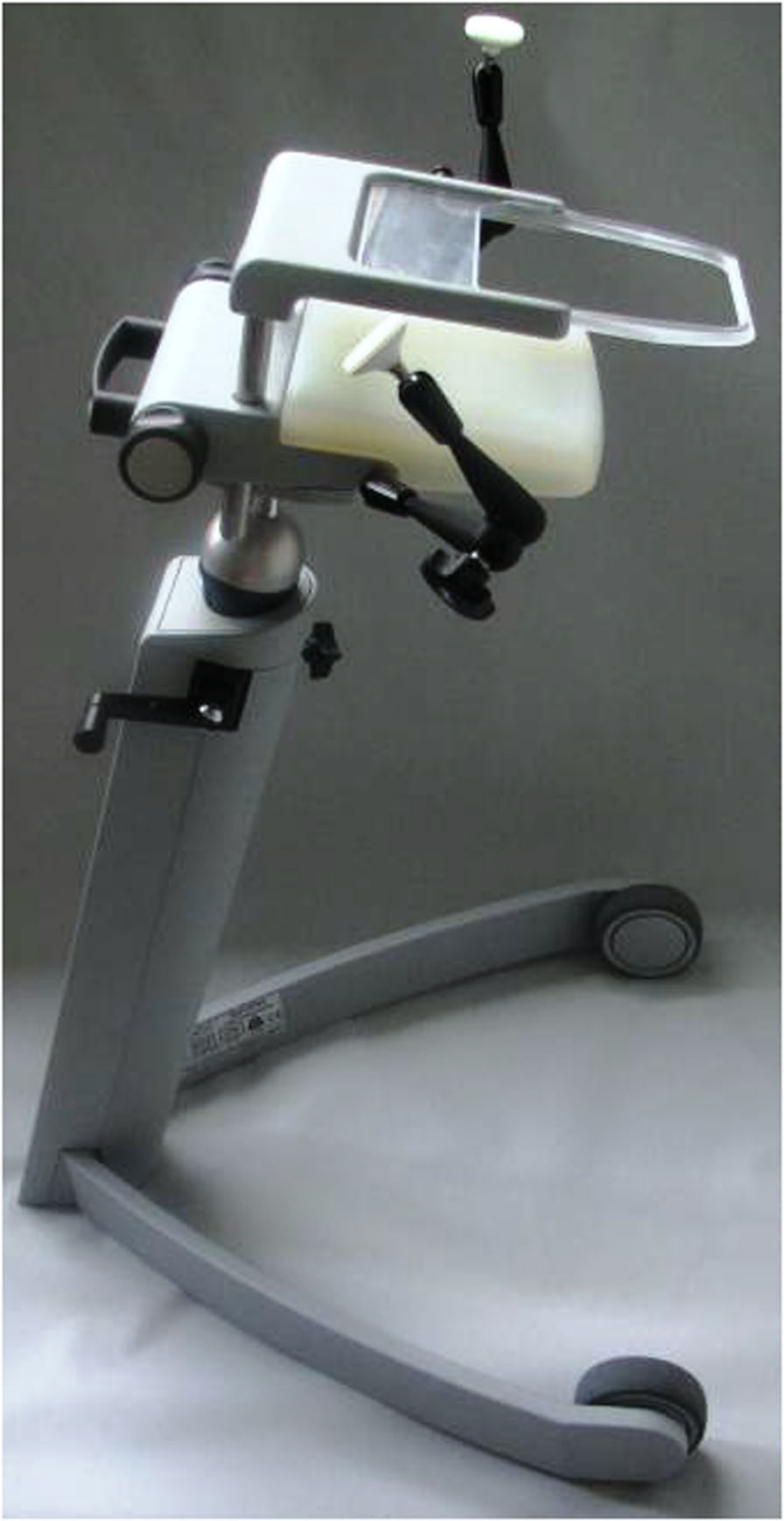
Breast support and immobilization system SenoPad (Theraclion, Paris, France)

The treatment was performed on an outpatient basis by a single physician (R.K.) with 7 years practice in US-guided HIFU therapy. The patient was placed in a lateral position to reduce the respiratory movements and the breast was supported by a special immobilization system (SenoPad, Theraclion, Paris, France), consisting in an adaptable plate with a silicon pad, a pressure plate and 2 articulated arms designed to fix the breast during the procedure (Fig. 1). Conscious intravenous analgesia was administered. The physician positioned the treatment head facing the targeted part of the breast and outlined the FA in two axes on the touch screen interface. Once the planning was finished, the treatment started with consecutive repeated HIFU pulses and cooling pauses with duration of 6 s and 54 s, respectively. The acoustic output power was 60 W and the intensity was 25 000 W/cm^2^. The treatment head moved automatically to cover the whole FA volume. The physician controlled the procedure and adjusted the applied energy to obtain the desired tissue reaction, characterized by hyperechoic marks as a sign of tissue damage [25]. When hyperechoic marks did not occur at maximal admissible energy, the treatment was continued at the lower energy level to avoid thermal damage of surrounding tissues. Treatment duration consisted of sonication, cooling and repositioning. During therapy, the patient’s vital parameters were monitored. In case of intolerant pain patients were asked to make a sign, in order to receive additional analgesics and the pause before the next pulse was extended with 30 to 60 s.

At the end of the HIFU procedure, pain related to the treatment was subjectively rated using a 0–100 mm visual analogue scale (VAS) and adverse events were assessed. After the 6-month follow-up, the patients completed a satisfaction questionnaire evaluating symptoms and cosmetic improvement with a 4-grade scale (1 – no, 2 – low, 3 – high, 4 – complete satisfaction).

### Data analysis

Data analysis was carried out using the Statistica software version 7.1 (StatSoft, Tulsa, OK, USA) and *p* < 0.05 was considered significant. Data are presented as mean ± standard deviation (SD) or median and range, as appropriate according to the distribution. To compare two groups, we applied the Student’s two-tailed *t*-test or Mann-Whitney *U*-test for continuous variables and the Chi-square for categorical variables. Comparisons of multiple variables were performed using ANOVA test, whereas for the follow-up comparison of longitudinally recorded data repeated measures ANOVA test was used. Correlations were calculated with the Pearson correlation test.

## Results

### Patient and FA characteristics

The mean age of 20 patients was 29.0 ± 10.2 years, 7/20 (35%) were previously operated for FA of the same breast and 7/20 (35%) had more than one FA. Nineteen of 26 FA (73.1%) were treated with one HIFU session (group 1). In 7/26 FA (26.9%) second HIFU ablation was performed (group 2) between 6 and 9 months (median 7 months) after the first session. FA in the group 2 were significantly larger at baseline, otherwise no significant differences in FA characteristics were observed between the two groups (Table 1).

**Table 1:** Baseline features of breast fibroadenomas treated with 1 session (group 1) or with 2 sessions (group 2) of HIFU

	Group 1	Group 2	*P* value
	(*n* = 19)	(*n* = 7)	
Age (years), mean ± SD	29.4 ± 10.8	26.6 ± 9.2	0.549^a^
BMI (kg/m^2^), mean ± SD	20.5 ± 3.4	20.0 ± 2.1	0.693^a^
Side, n (%)			
left	6 (31.6)	3 (42.9)	0.945^b^
right	13 (68.4)	4 (57.1)	
Quadrant, n (%)			
up-out	8 (42.1)	2 (28.6)	0.454^b^
up-in	6 (31.6)	3 (42.9)	
low-in	3 (15.8)	0 (0.0)	
low-out	2 (10.5)	2 (28.6)	
Depth (mm), mean ± SD	15.3 ± 3.9	15.9 ± 3.6	0.710^a^
Basal volume (mL), median (range)	1.82 (0.35–5.95)	8.14 (1.53–10.39)	0.0140^c^
Color flow Doppler pattern (%)			
0	8 (42.1)	2 (28.5)	0.194^b^
I	8 (42.1)	1 (14.3)	
II	2 (10.5)	3 (42.9)	
III	1 (5.3)	1(14.3)	

^a^Student’s *t*-test, ^b^Chi-square-test, ^c^Mann-Whitney *U* test

During the first HIFU session, the treated volume, total applied energy, treatment duration and the number of treated sites were significantly higher in group 2 than in group 1. The percentage of the treated volume was significantly lower in group 2, whereas the energy per treated volume and the percentage of hyperechoic marks did not differ between both groups (Table 2). During the second HIFU session, the treated volume and total applied energy in group 2 were still larger compared with the baseline values in group 1, whereas other parameters did not differ significantly (Table 2).

**Table 2:** Treatment characteristics at each HIFU session

	Group 1 (*n* = 19)	Group 2 (*n* = 7)	
	1^st^ HIFU	1^st^ HIFU	2^nd^ HIFU
Treated volume (mL), median (range)	0.78 (0.35–2.24)^a^	2.66 (0.52–3.01)	1.34 (0.65–2.24)
Treated volume (%), mean ± SD	56.96 ± 25.05	36.02 ± 7.92^b^	58.50 ± 22.34
Total delivered energy (kJ), median (range)	10.1 (4.4–25.4)^c^	27.7 (7.4–39.6)	16.5 (12.8–31.4)
Energy per treated volume (kJ/mL), median, range	12.4 (6.7–14.3)	13.0 (10.4–14.2)	13.9 (7.1–14.8)
Treatment duration (min) mean ± SD	60.6 ± 22.8^d^	105.1 ± 38.8	66.3 ± 15.7
Number of treated sites, median (range)	58 (29–149)^e^	179 (38–221)	95 (40–153)
Hyperechoic marks (%), median (range)	15 (0–31)	10 (0–29)	7 (0–50)

^a^ *p* < 0.05 compared with group 2 at the 1^st^ HIFU and group 2 at the 2^nd^ HIFU, Mann-Whitney *U* test, ^b^ *p* < 0.05 compared with group 1 and group 2 at the 2^nd^ HIFU, Student’s *t*-test, ^c^ *p* < 0.01 compared with group 2 at the 1^st^ HIFU and group 2 at the 2^nd^ HIFU, Mann-Whitney *U* test, ^d^ *p* < 0.001 compared with group 2 at the 1^st^ HIFU, Student’s *t*-test, ^e^ *p* < 0.05 compared with group 2 at the 1^st^ HIFU, Mann-Whitney *U* test

### FA volume reduction

**Fig. 2:**
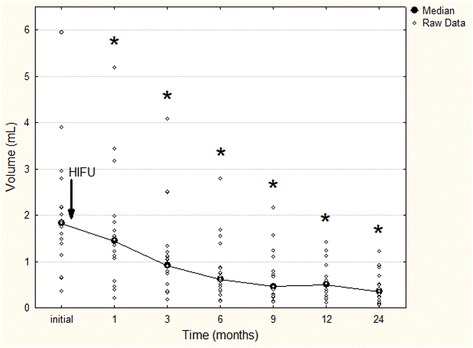
Fibroadenoma volume reduction in patients treated with one HIFU session. *p < 0.001 compared with the initial value before treatment (repeated measures analysis of variance test)

**Fig. 3:**
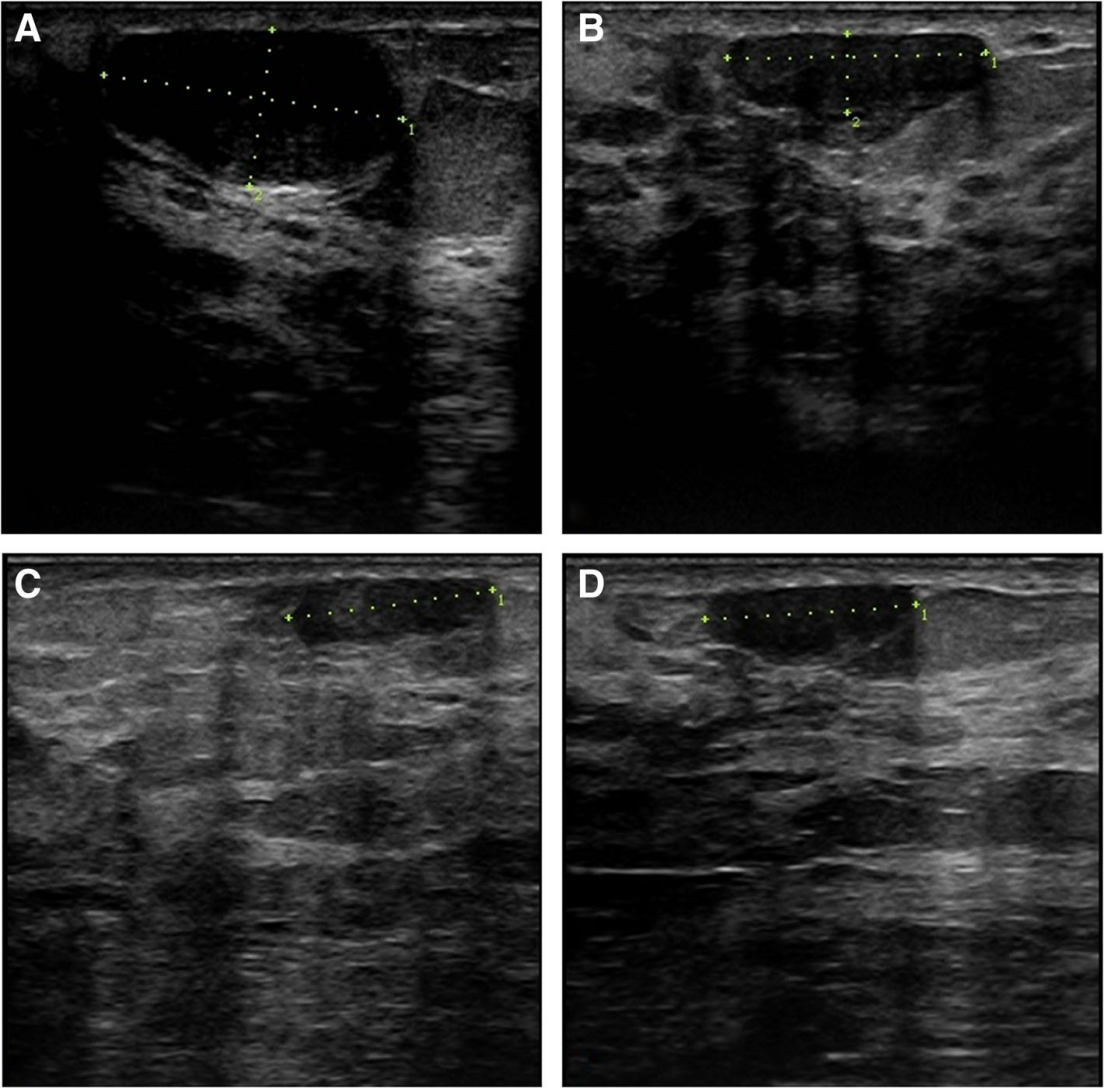
Radial scan of left breast fibroadenoma in 27-years old woman treated with one HIFU session. **a** baseline US shows an oval-shaped hypoechoic well-defined lesion of 1.87 ml of volume; **b** 6 months after the treatment 64.6% of volume reduction was found; **c** at 12-month follow-up the volume reduction was 73%; **d** the tendency continued up to 24 months with 78.6% of total volume reduction

**Fig. 4:**
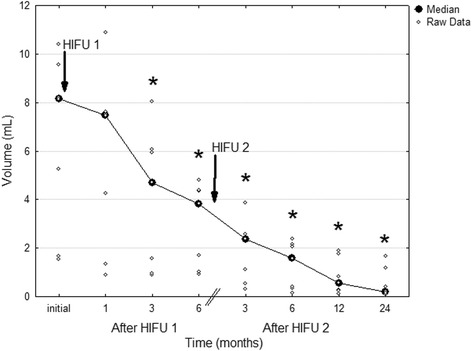
Fibroadenoma volume reduction in patients treated with two HIFU sessions. *p < 0.01 compared with the initial value before treatment (repeated measures analysis of variance test)

**Fig. 5:**
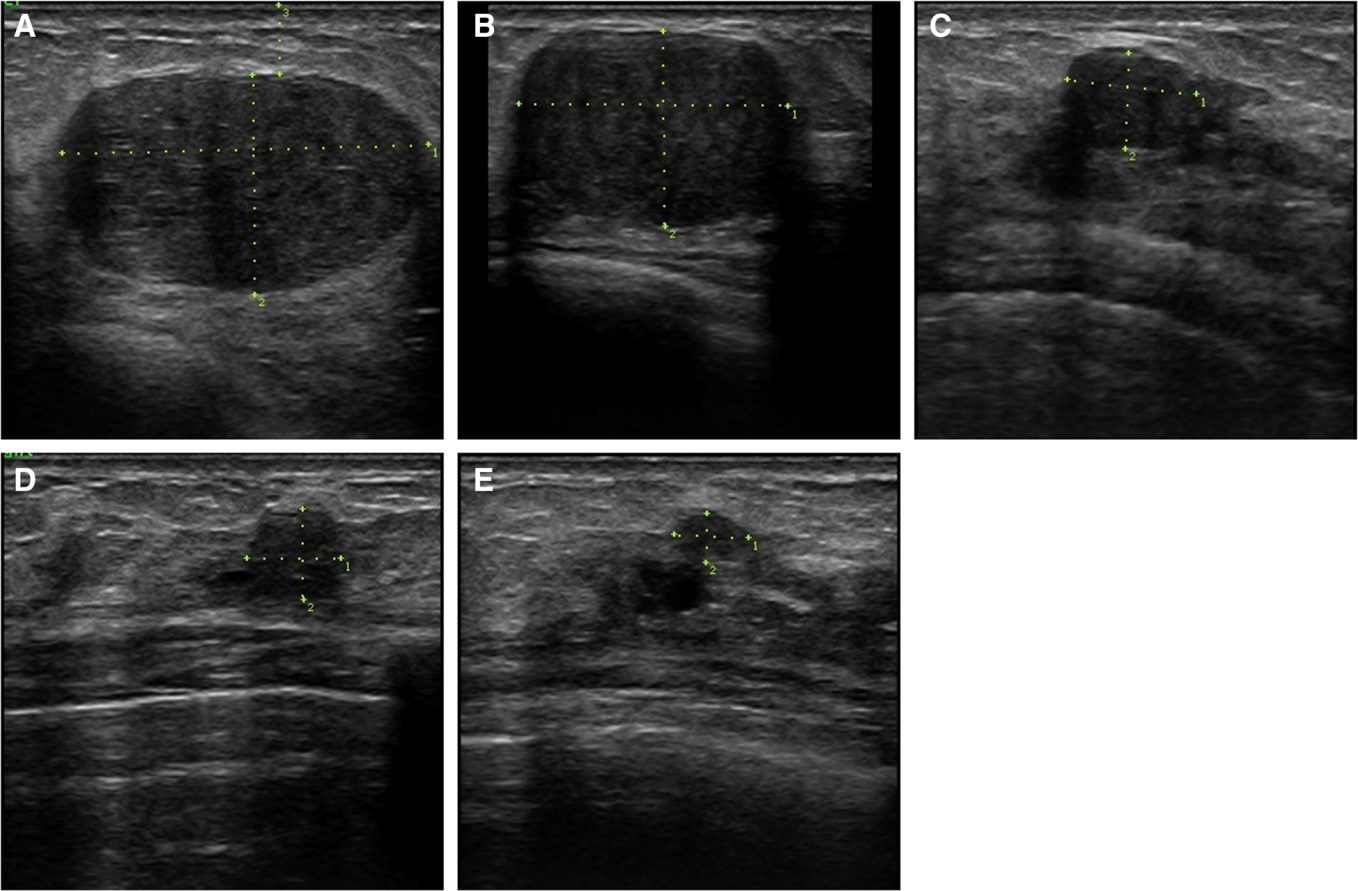
Anti-radial scan of right breast fibroadenoma in 39-years old woman treated with two HIFU sessions. **a** baseline US shows an oval-shaped hypoechoic well-defined lesion with volume of 10.87 ml; **b** 6 months after the first HIFU ablation 58.2% of volume reduction was observed, but the FA was still large (4.35 ml); **c** 6 months after the second HIFU ablation the volume reduction was 96.5% from baseline; **d** the reduction progressed at 12 months up to 98.7%; **e** at 24-month follow-up the total volume reduction was 99.1%

In group 1, FA volume decreased significantly as soon as at 1-month follow-up (median 1.44 mL, range 0.21–5.18 mL, *p* < 0.001 compared with the initial value), and continued to reduce until the 24-month visit (median 0.35 mL, range 0.06–1.21 mL; *p* < 0.001 compared with the initial value) (Fig. 2). Illustration of FA treated with one HIFU session is shown in Fig. 3. In group 2, a significant FA shrinkage was observed at 3-month follow-up after the first HIFU session (median 4.70 mL, range 0.88–8.02 mL, *p* = 0.005 compared with the initial value). After the second HIFU session, the significant volume reduction continued until the 24-month visit (median 0.21 mL, range 0.09–1.66 mL, *p* = 0.003 compared with the initial value) (Fig. 4). Reduction of FA treated with two HIFU sessions is demonstrated in Fig. 5.

The percentage of volume reduction was comparable between both groups until 6-month follow-up after the first HIFU (mean 58.04 ± 16.9% in group 1 and 50.44 ± 10.38% in group 2, respectively). However, at 12-month visit after the final treatment the percentage of volume reduction was significantly higher in group 2 that received two treatments (mean 71.02 ± 10.39% in group 1 compared with 86.28 ± 7.64% in group 2, *p* = 0.002). Similar difference was found at 24-month visit (mean 77.32 ± 13.47% in group 1 compared with 90.47 ± 7.13% in group 2, *p* = 0.025), when the maximal volume reduction in group 1 and 2 reached 94.67% and 99.13%, respectively. During 24-month follow-up no case of regrowth was observed.

In order to estimate the effects of HIFU on FA shrinkage, we further compared the reduction of individual diameters at 6-month follow-up after the first HIFU. In all FA, the percentages of *d1*, *d2* and *d3* reduction were comparable (mean 26.44% ± 12.64%, 25.92 ± 11.44%, 19.99 ± 24.22%, respectively). After HIFU therapy, color Doppler flow decreased or totally disappeared. We found a significant positive correlation of initial CFD pattern with FA volume reduction at 12 months (*r* = 0.450, *p* = 0.021).

### Safety, tolerability and satisfaction of patients

HIFU was well tolerated by all patients and no serious adverse events were observed. The VAS score at the first HIFU session (mean 40.7 ± 24.6, range 5–78) did not differ significantly from the VAS score at the second session (mean 34.9 ± 17.9, range 7–64). There was no significant correlation between the VAS score and age, BMI, FA depth, total applied energy as well as the applied energy per volume, initial FA volume or the duration of treatment. Up to one week after the first treatment, 9/20 patients (45.0%) reported about mild to moderate pain or tenderness of the treated FA, and similar sensation was reported in 4/7 (57.1%) patients after the second session. No patient needed additional analgesic drugs after the therapy.

During the first HIFU treatment, 4 patients developed mild subcutaneous oedema that disappeared at 1 week without therapy. Immediately after the treatment, mild to moderate erythema was detected in 3 patients treated twice. In 2 of them, this reaction disappeared up to 1 week after each HIFU session. Only in one patient with BMI 17.4 kg/m^2^ it evoluted as first-degree skin burn with crusts and hyperpigmentation visible up to 6-month follow-up. All side effects were transient and showed complete resolution.

### Patients' satisfaction

All patients completed a questionnaire. The level of satisfaction with symptom disappearance was high (grade 3) in 50%, 45% were satisfied completely (grade 4), whereas in 1 patient (5%) the level of satisfaction was low (grade 2). With respect to cosmetic results, 19 out of 20 patients (95%) were satisfied completely (grade 4) and in 1 patient (5%) the level of satisfaction was high.

## Discussion

Our single-center study demonstrates that US-guided HIFU treatment is effective in treating breast FA as indicated by a significant and stable volume reduction at two years follow-up, when the mean reduction was 77.32% after one HIFU session and 90.47% after two HIFU sessions. The procedure is safe, with favourable cosmetic outcome and patient’s satisfaction.

The significant reduction of FA volume as a result of US-guided HIFU is consistent with a report based on multicentre study, where 72.5% FA volume reduction was shown at 1-year follow-up [23]. A favourable treatment effect of HIFU was previously described in patients with breast cancer, in whom coagulation necrosis has been obtained in 78% to 100% of treated breast tumour volume [26, 27]. Based on our data, HIFU ablation results in substantial FA volume reduction 1 month after therapy, and the volume continues to regress during the long-term follow-up. According to this stable tendency, further reduction may be expected. Besides, our data show that with second treatment the volume reduction can be additionally enhanced.

Feasibility and efficacy of HIFU performed under MR guidance were first demonstrated in breast FA more than a decade ago [21]. Compared with MR guidance, US-guided FA treatment is faster, more comfortable for the patient and it does not need contrast agent application [28]. Besides, it enables real-time visualization of the targeted volume as well as apparent grayscale changes during the treatment for monitoring the response to HIFU, demonstrated also in other treatment indications [20, 29].

Currently, the accepted definitive treatment of FA is breast-conserving surgery which removes the FA entirely with a subsequent tissue volume defect and risk of complications as bleeding, seroma formation and chronic incisional pain [30]. The minimally invasive US-guided vacuum-assisted percutaneous excision has better cosmetic results but incomplete removal with 3.4% to 30% rate of residual tumour associated with initial tumour size over 20 mm [13, 31]. HIFU as an ablative treatment modality competes with up-to-date established ablation methods such as radiofrequency, laser or cryoablation. However, the US-guided HIFU is entirely non-invasive, without need of probe or antenna insertion in the FA. It requires less recovery time and cost, and is associated with only minor adverse events and with no complications as hematoma, infection or scar formation [21, 26]. It can be performed in case of multiple lesions, in one or both breasts. During the long-term follow-up of our patients, the residual lesions did not demonstrate a potential for regrowth, which cannot be stated for still viable residual tissue after vacuum-assisted percutaneous excision [31]. When the results are not satisfactory after one treatment or the FA is still large, second HIFU is a good option as shown in our study.

An important drawback of the HIFU treatment is the long duration, which increases with the size of treated FA. Another limitation is one layer treatment, which may result in incomplete ablation. However, the later presumption is not supported by our observation of proportionate reduction of all three FA diameters, but larger studies are needed to evaluate the exact mechanism of FA shrinkage after ablation. Besides, an appropriate patient selection is needed in order to assure the accessibility of the targeted FA. Whereas deeply situated lesion may not be accessible to US beam, the superficial FA localization associated with scarcity of subcutaneous fat may result in skin burn as observed in one of our patients with low BMI. Certainly, the main limitation of the study is the small number of observed patients, especially in the group treated with two sessions, which is mainly due to the recent introduction of the method and lack of previous experience.

## Conclusions

Our results of US-guided HIFU ablation of breast FA provide evidence that the method is promising, efficient and safe. Although one treatment session results in marked reduction of FA volume, the second treatment significantly increases the degree of volume reduction. Due to its non-invasive nature, the method is well tolerated and associated with high level of patients’ satisfaction. Therefore, US-guided HIFU may become an alternative to other well-established treatment modalities of breast FA. However, larger cohorts of patients with similar or even longer follow-up should be evaluated in order to define the treatment success.

## Abbreviations

BI-RADS: Breast imaging and reporting data system
BMI: Body mass index
CFD: Colour flow Doppler
FA: Fibroadenoma
HIFU: High-intensity focused ultrasound
MR: Magnetic resonance
SD: Standard deviation
US: Ultrasound
VAS: Visual analog scale.

## References

[1] Olu-Eddo AN, Ugiagbe EE (2011). Benign breast lesions in an African population: A 25-year histopathological review of 1864 cases. Niger Med J..

[2] Aslam HM, Saleem S, Shaikh HA, Shahid N, Mughal A, Umah R (2013). Clinico- pathological profile of patients with breast diseases. Diagn Pathol..

[3] Greenberg R, Skornick Y, Kaplan O (1998). Management of breast fibroadenomas. J Gen Intern Med..

[4] Masciadri N, Ferranti C (2011). Benign breast lesions: Ultrasound. J Ultrasound..

[5] Guray M, Sahin AA (2006). Benign breast diseases: classification, diagnosis, and management. Oncologist..

[6] Santen RJ, Mansel R (2005). Benign breast disorders. N Engl J Med..

[7] Dixon JM, Dobie V, Lamb J, Walsh JS, Chetty U (1996). Assessment of the acceptability of conservative management of fibroadenoma of the breast. Br J Surg..

[8] Carter BA, Page DL, Schuyler P, Parl FF, Simpson JF, Jensen RA (2001). No elevation in long-term breast carcinoma risk for women with fibroadenomas that contain atypical hyperplasia. Cancer..

[9] Worsham MJ, Raju U, Lu M, Kapke A, Botttrell A, Cheng J (2009). Risk factors for breast cancer from benign disease in a diverse population. Breast Cancer Res Treat..

[10] Sklair-Levy M, Sella T, Alweiss T, Craciun I, Libson E, Mally B (2008). Incidence and management of complex fibroadenomas. AJR Am J Roentgenol..

[11] Harvey JA, Nicholson BT, Lorusso AP, Cohen MA, Bovbjerg VE (2009). Short-term follow-up of palpable breast lesions with benign imaging features: evaluation of 375 lesions in 320 women. AJR Am J Roentgenol..

[12] Anderson BO, Masetti R, Silverstein MJ (2005). Oncoplastic approaches to partial mastectomy: an overview of volume displacement techniques. Lancet Oncol..

[13] Grady I, Gorsuch H, Wilburn-Bailey S (2008). Long-term outcome of benign fibroadenomas treated by ultrasound-guided percutaneous excision. Breast J..

[14] Thurley P, Evans A, Hamilton L, James J, Wilson R (2009). Patient satisfaction and efficacy of vacuum-assisted excision biopsy of fibroadenomas. Clin Radiol..

[15] Hahn M, Pavlista D, Danes J, Klein R, Golatta M, Harcos A (2013). Ultrasound guided cryoablation of fibroadenomas. Ultraschall Med..

[16] Lai LM, Hall-Craggs MA, Mumtaz H, Ripley PM, Davidson TI, Kissin MW (1999). Interstitial laser photocoagulation for fibroadenomas of the breast. Breast..

[17] Fornage BD, Sneige N, Ross MI, Mirza AN, Kuerer HM, Edeiken BS (2004). Small (< or = 2-cm) breast cancer treated with US-guided radiofrequency ablation: feasibility study. Radiology..

[18] Vargas HI, Dooley WC, Gardner RA, Gonzales KD, Venegas R, Heywang-Kobrunner SH (2004). Focused microwave phased array thermotherapy for ablation of early-stage breast cancer: results of thermal dose escalation. Ann Surg Oncol..

[19] Zhou W, Zha X, Liu X, Ding Q, Chen L, Ni Y (2012). US-guided percutaneous microwave coagulation of small breast cancers: a clinical study. Radiology..

[20] Zhou YF (2011). High intensity focused ultrasound in clinical tumor ablation. World J Clin Oncol..

[21] Hynynen K, Pomeroy O, Smith DN, Huber PE, McDannold NJ, Kettenbach J (2001). MR imaging–guided focused ultrasound surgery of fibroadenomas in the breast: a feasibility study. Radiology..

[22] Peek MCL, Ahmed M, Scudder J, Baker R, Pinder SE, Douek M (2016). High intensity focused ultrasound in the treatment of breast fibroadenomata: results of the HIFU-F trial. Int J Hyperthermia.

[23] Kovatcheva R, Guglielmina JN, Abehsera M, Boulanger L, Laurent N, Poncelet E (2015). Ultrasound-guided high-intensity focused ultrasound treatment of breast fibroadenoma-a multicenter experience. J Ther Ultrasound..

[24] Vitti P, Rago T, Mazzeo S, Brogioni S, Lampis M, De Liperi A (1995). Thyroid blood flow evaluation by color-flow Doppler sonography distinguishes Graves' disease from Hashimoto's thyroiditis. J Endocrinol Invest.

[25] Rabkin BA, Zderic V, Crum LA, Vaezy S (2006). Biological and physical mechanisms of HIFU-induced hyperecho in ultrasound images. Ultrasound Med Biol..

[26] Furusawa H, Namba K, Thomsen S, Akiyama F, Bendet A, Tanaka C (2006). Magnetic resonance-guided focused ultrasound surgery of breast cancer: reliability and effectiveness. J Am Coll Surg..

[27] Merckel LG, Knuttel FM, Deckers R, van Dalen T, Shubert G, Peters NH (2016). First clinical experience with a dedicated MRI-guided high-intensity focused ultrasound system for breast cancer ablation. Eur Radiol..

[28] Marincola BC, Pediconi F, Anzidei M, Miglio E, Di Mare L, Telesca M (2015). High-intensity focused ultrasound in breast pathology: non-invasive treatment of benign and malignant lesions. Expert Rev Med Devices..

[29] Orsi F, Arnone P, Chen W, Zhang L (2010). High intensity focused ultrasound ablation: a new therapeutic option for solid tumors. J Cancer Res Ther..

[30] Nyström L, Andersson I, Bjurstam N, Frisell J, Nordenskjöld B, Rutqvist LE (2002). Long-term effects of mammography screening: updated overview of the Swedish randomized trials. Lancet..

[31] Wang WJ, Wang Q, Cai QP, Zhang JQ (2009). Ultrasonographically guided vacuum-assisted excision for multiple breast masses: non-randomized comparison with conventional open excision. J Surg Oncol..

